# Supplementary material for: Locityper enables targeted genotyping of complex polymorphic genes
Source: Nat Genet. 2025 Oct 17;57(11):2901–8. doi: 10.1038/s41588-025-02362-4 (PMC12597825; doi:10.1038/s41588-025-02362-4)
Supplement: Supplementary file 1 — Supplementary Figs. 1–3 and information. [file 41588_2025_2362_MOESM1_ESM.pdf]

nature genetics

Supplementary information https://doi.org/10.1038/s41588-025-02362-4

Locityper enables targeted genotyping of complex polymorphic genes

In the format provided by the authors and unedited

1 **Supplementary Figures** **2**

Supplementary Figure 1. Locityper haplotyping accuracy (LOO) . . . 2

Supplementary Figure 2. Locityper haplotyping accuracy (full) . . . 3

Supplementary Figure 3. Variant call set accuracy . . . 4

2 **Supplementary Information** **5**

2.1 Variant call set comparison . . . 5

2.2 Estimating Negative Binomial parameters . . . 5

2.3 Alternative Integer Linear Programming formulation . . . 6

1 Supplementary Figures

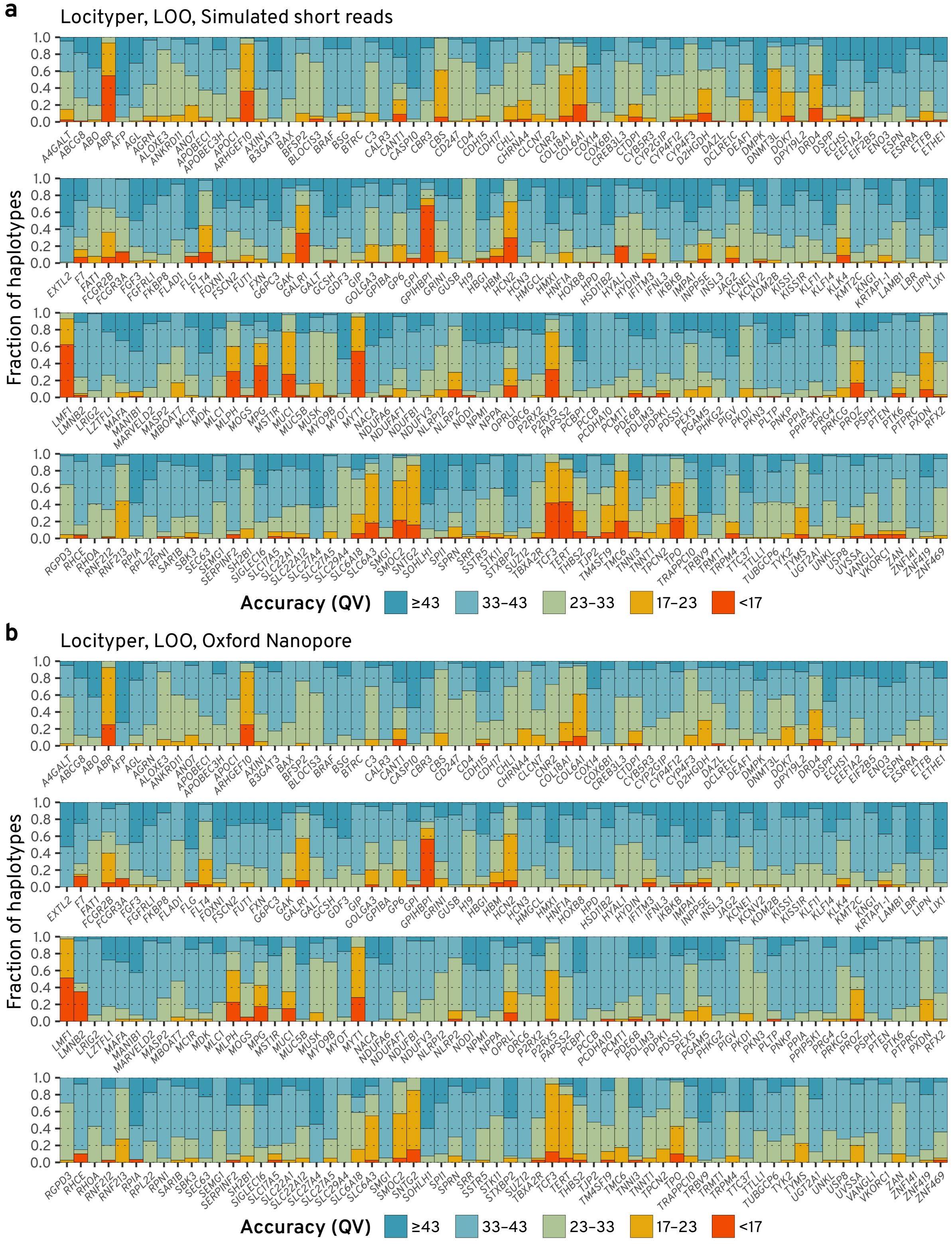

Supplementary Figure 1. Locityper haplotyping accuracy across 256 challenging medically relevant loci in leave-one-out configuration. Haplotypes are stratified into five bins based on their quality values (QV; shown with different colors). **a**, Locityper accuracy at 44 simulated short-read datasets. **b**, Locityper accuracy at 20 Oxford Nanopore datasets.

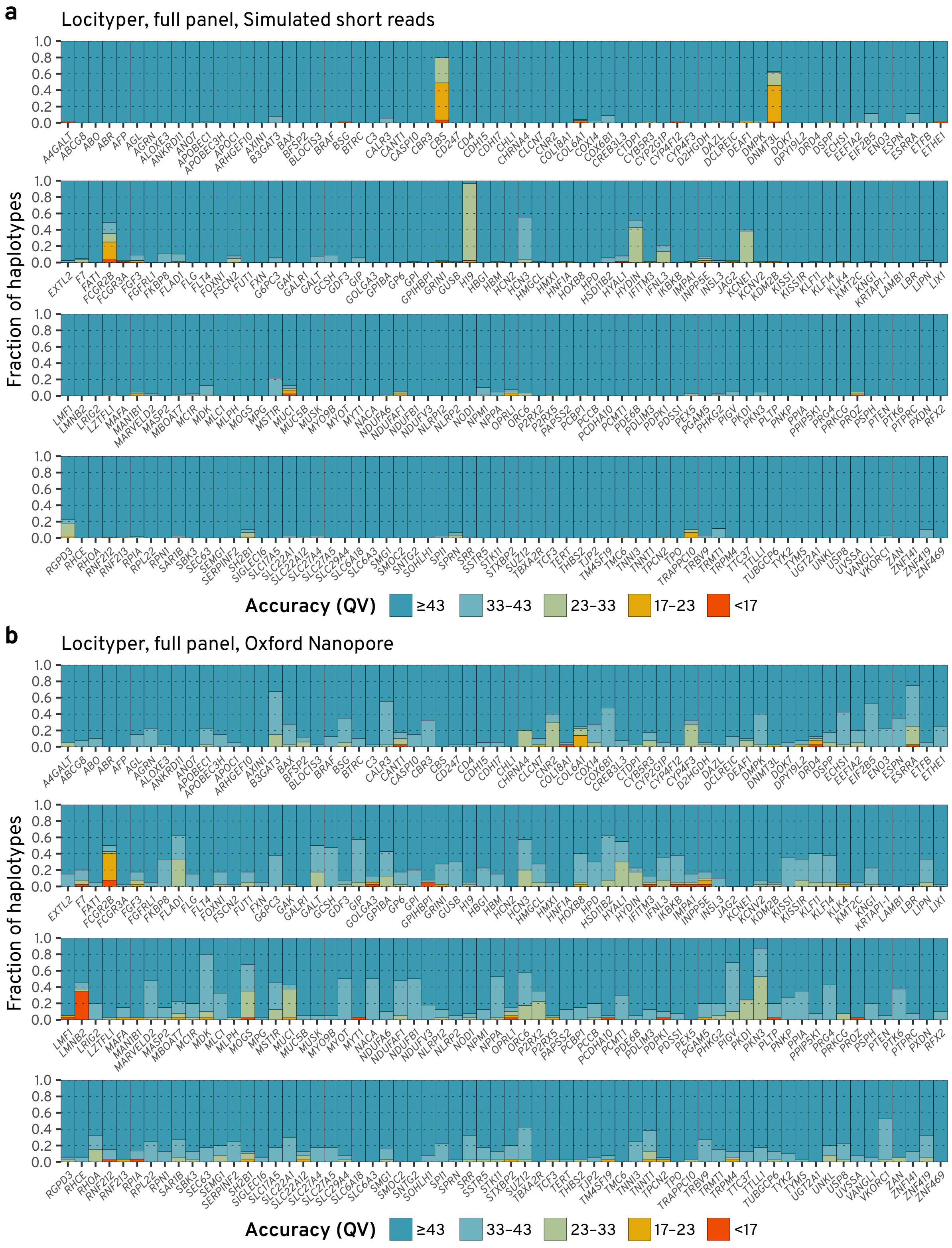

Supplementary Figure 2. Locityper haplotyping accuracy across 256 challenging medically relevant loci using full reference panel. a, Locityper accuracy at 44 simulated short-read datasets. **b**, Locityper accuracy at 20 Oxford Nanopore datasets.

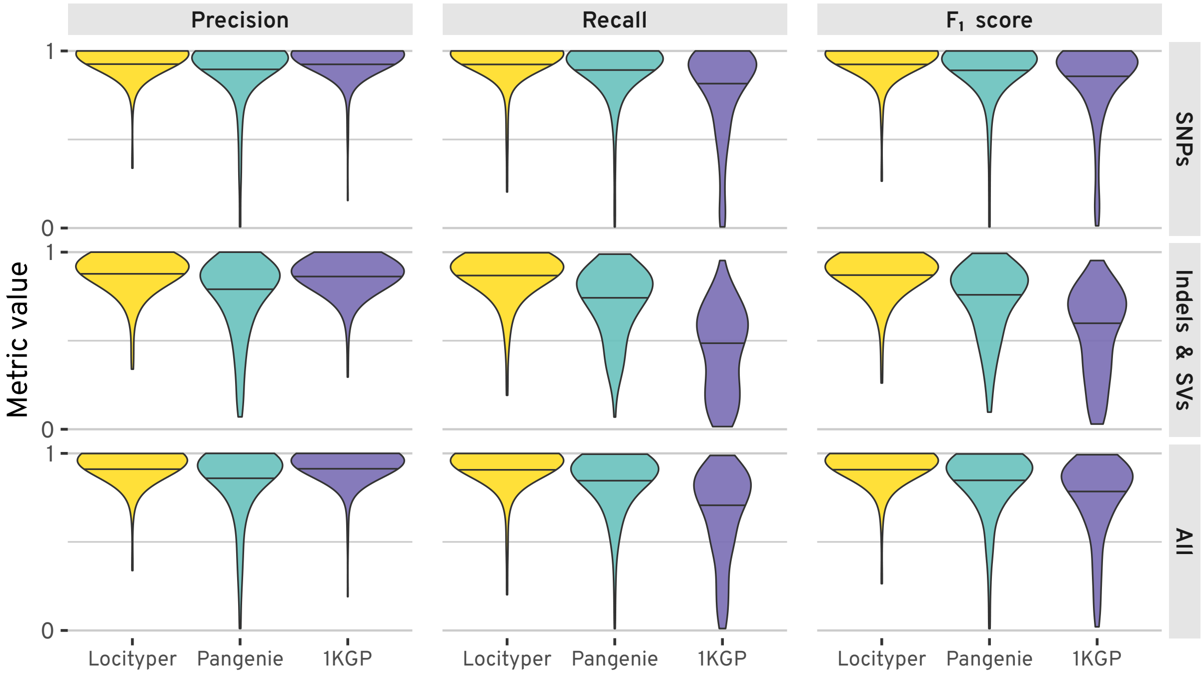

Supplementary Figure 3. Variant call set accuracy for Locityper, Pangenie, and the 1KGP variant call sets across 256 medically relevant loci. The figure shows variant calling precision, recall and F_1 scores, stratified by the variant type (SNPs; indels and SVs; and all together). Black horizontal lines show median values across all loci. Locityper and Pangenie were run using their full respective reference panels, which include input datasets.

2 Supplementary Information

2.1 Variant call set comparison

Pangenie is a pangenome-based short read variant caller, which calls sequence variants and structural variations by counting read k-mers along paths in a pangenomic graph[1]. In order to facilitate comparison to unphased Pangenie call sets, we converted Locityper-predicted locus haplotypes into phased diploid variant calls, and compared Pangenie and Locityper call sets to the ground truth variant calls, extracted from the phased whole genome assemblies for the 40 HPRC samples. Both Locityper and Pangenie were run using their full respective reference panels, which include HPRC samples.

To compare Locityper `v0.15.1`, Pangenie `v3.02`, and 1KGP call sets, we decomposed and normalized variant calls using Vt[2] `v0.57721` commands `decompose_blocksub` and `normalize`, respectively. Then, we used RTG tools[3] `v3.12.1` `vcfeval` module to calculate variant calling precision and recall using existing HPRC Minigraph-cactus[4] representation as a baseline call set.

Across the 256 CMR loci, Locityper achieves higher average F_1 score (0.939, median = 0.965) than Pangenie (0.842, median = 0.902), improving both average precision (Pangenie: 0.848, Locityper: 0.945); and recall (Pangenie: 0.843, Locityper: 0.936) by over 9% (see Supp. Figure 3). Although 1KGP call set shows similarly high precision (0.946), it does so at the expense of much lower recall (0.658), achieving a combined F_1 score of 0.746 (median = 0.829). The difference is more pronounced at indels and structural variations, where Locityper, Pangenie and 1KGP call sets attain average F_1 scores of 0.882, 0.728 and 0.550, respectively.

Over the full genome, Pangenie shows very high genotyping accuracy; however, its reliance on unique k-mers results in lower calling power at especially challenging loci. We envision that genome-wide Pangenie variant calling can be complemented by targeted Locityper analysis to produce scalable and accurate genome-wide variant calling workflow.

2.2 Estimating Negative Binomial parameters

Negative Binomial (NB) parameters n and ψ can easily be calculated based on the sample mean m and variance v using the method of moments:

$$n = \frac{m^2}{v - m}, \quad \psi = \frac{m}{v}. \tag{1}$$

However, in cases when the underlying distribution is similar to the Poisson distribution, observed variance v can become very similar to mean m (or even smaller than m). In such cases, parameter n can become very large, or even negative (forbidden under NB definition). For these reasons, we employ L_1 regularization on n.

If input reads were subsampled with rate s (also known as s-thinning), distribution $\mathrm{NB}(n, \psi)$ is transformed[5,6] into distribution $\mathrm{NB}\left(n, \frac{\psi}{s+\psi-s\psi}\right)$. Accordingly, we correct read depth distributions according to the subsampling rate s, used during WGS dataset preprocessing. To summarize, we estimate NB parameters n and ψ based on sample mean m, variance v and subsampling rate s:

$$\underset{\substack{n>0 \\ \psi\in(0,1)}}{\operatorname{argmin}} \left(\frac{ns\cdot(1-\psi)}{\psi} - m\right)^2 + \left(\frac{ns\cdot(1-\psi)\cdot(\psi+s-\psi s)}{\psi^2} - v\right)^2 + \lambda n, \tag{2}$$

where λ is the regularization parameter (10^{-5} by default).

2.3 Alternative Integer Linear Programming formulation

Theoretically, locus genotyping problem can be stated in a single ILP statement. For a given ploidy π let us define an integer variable $q_a \in \{0, \ldots, \pi\}$ for each haplotype $a \in A$. Then, we can generalize the problem statement from the main text in the following way:

$$\begin{aligned}
&\text{Maximize} \quad \Omega_R \sum_{\mathbf{r}\in R} \sum_{a\in A} \sum_{\mathbf{w}\in L^{(a)}} x_{\mathbf{rw}} \cdot \log \mathcal{P}_{\mathbf{rw}} + \Omega_D \sum_{a\in A} \sum_{w\in W^{(a)}} \sum_{d=0}^{D_{\max}} y_{wd} \cdot \zeta_w \cdot \varphi_w(d) \\
&\text{Subject to} \quad \sum_{a\in A} q_a = \pi, \\
&\sum_{a\in A} \sum_{\mathbf{w}\in L^{(a)}} x_{\mathbf{rw}} = 1 \qquad \forall \mathbf{r} \in R, \\
&\sum_{d=0}^{D_{\max}} y_{wd} = q_a \qquad \forall a \in A,\ \forall w \in W^{(a)}, \\
&\sum_{\mathbf{r}\in R} \sum_{u\in W^{(\mathbf{g})}} \left(x_{\mathbf{r},wu} + x_{\mathbf{r},uw}\right) - \sum_{d=0}^{D_{\max}} d \cdot y_{wd} = 0 \qquad \forall a \in A,\ \forall w \in W^{(a)}, \\
&\text{and} \quad x_\diamond \in \{0, 1\}, \\
&y_\diamond \in \{0, \ldots, \pi\}, \\
&q_\diamond \in \{0, \ldots, \pi\}.
\end{aligned} \tag{3}$$

Even though this formulation is very flexible and allows for higher ploidy, the large number of variables and complex interactions between them makes the problem almost infeasible for state-of-the-art ILP solvers. Both Gurobi[7] and HiGHS[8] required significantly more time to solve the problem and reached worse likelihoods, compared to the sum time required to solve the ILP problem for each of the locus genotypes. Additionally, generalized problem statement does not allow to directly estimate genotype likelihood for non-primary genotype predictions.

References

1. Ebler, J. *et al.* Pangenome-based genome inference allows efficient and accurate genotyping across a wide spectrum of variant classes. *Nature Genetics* **54,** 518–25 (2022).
2. Tan, A., Abecasis, G. R. & Kang, H. M. Unified representation of genetic variants. *Bioinformatics* **31,** 2202–4 (2015).
3. Cleary, J. G. *et al.* Comparing variant call files for performance benchmarking of next-generation sequencing variant calling pipelines. *BioRxiv,* 023754 (2015).
4. Hickey, G. *et al.* Pangenome graph construction from genome alignments with Minigraph-Cactus. *Nature Biotechnology,* 1–11 (2023).
5. Wiuf, C. & Stumpf, M. P. Binomial subsampling. *Proceedings of the Royal Society A* **462,** 1181–1195 (2006).
6. Levina, A. & Priesemann, V. Subsampling scaling. *Nature Communications* **8,** 15140 (2017).
7. Gurobi Optimization. *Gurobi optimizer reference manual* `https://www.gurobi.com`. 2023.
8. Huangfu, Q. & Hall, J. J. Parallelizing the dual revised simplex method. *Mathematical Programming Computation* **10,** 119–142 (2018).
