# Supplementary material for: Locityper enables targeted genotyping of complex polymorphic genes
Source: Nat Genet. 2025 Oct 17;57(11):2901–8. doi: 10.1038/s41588-025-02362-4 (PMC12597825; doi:10.1038/s41588-025-02362-4)
Supplement: Supplementary file 2 — Reporting Summary [file 41588_2025_2362_MOESM2_ESM.pdf]

nature portfolio

Corresponding author(s): Timofey Prodanov, Tobias Marschall

Last updated by author(s): 14.07.2025

Reporting Summary

Nature Portfolio wishes to improve the reproducibility of the work that we publish. This form provides structure for consistency and transparency in reporting. For further information on Nature Portfolio policies, see our Editorial Policies and the Editorial Policy Checklist.

Statistics

For all statistical analyses, confirm that the following items are present in the figure legend, table legend, main text, or Methods section.

n/a	Confirmed	
☐	☒	The exact sample size (*n*) for each experimental group/condition, given as a discrete number and unit of measurement
☒	☐	A statement on whether measurements were taken from distinct samples or whether the same sample was measured repeatedly
☐	☒	The statistical test(s) used AND whether they are one- or two-sided<br>*Only common tests should be described solely by name; describe more complex techniques in the Methods section.*
☒	☐	A description of all covariates tested
☒	☐	A description of any assumptions or corrections, such as tests of normality and adjustment for multiple comparisons
☐	☒	A full description of the statistical parameters including central tendency (e.g. means) or other basic estimates (e.g. regression coefficient) AND variation (e.g. standard deviation) or associated estimates of uncertainty (e.g. confidence intervals)
☐	☒	For null hypothesis testing, the test statistic (e.g. *F*, *t*, *r*) with confidence intervals, effect sizes, degrees of freedom and *P* value noted<br>*Give P values as exact values whenever suitable.*
☒	☐	For Bayesian analysis, information on the choice of priors and Markov chain Monte Carlo settings
☒	☐	For hierarchical and complex designs, identification of the appropriate level for tests and full reporting of outcomes
☒	☐	Estimates of effect sizes (e.g. Cohen's *d*, Pearson's *r*), indicating how they were calculated

Our web collection on statistics for biologists contains articles on many of the points above.

Software and code

Policy information about availability of computer code

Data collection	ART Illumina v2.5.8
Data analysis	Locityper v0.18.0 (https://github.com/tprodanov/locityper, https://zenodo.org/records/14861388), Pangenie v3.02, T1K v1.0.5, Jellyfish v2.2.10, Minimap2 v2.26-r1175, Strobealign v0.13.0, Samtools v1.21, Bcftools v1.21, Tabix v1.21, Vt v0.57721, RTG-tools v3.12.1, Immuannot e8da19c

For manuscripts utilizing custom algorithms or software that are central to the research but not yet described in published literature, software must be made available to editors and reviewers. We strongly encourage code deposition in a community repository (e.g. GitHub). See the Nature Portfolio guidelines for submitting code & software for further information.

Data

Policy information about availability of data

All manuscripts must include a data availability statement. This statement should provide the following information, where applicable:

- Accession codes, unique identifiers, or web links for publicly available datasets
- A description of any restrictions on data availability
- For clinical datasets or third party data, please ensure that the statement adheres to our policy

Locityper-predicted genotypes for 3202 Illumina 1KGP samples, corresponding preprocessed WGS parameters, target loci database, simulation seeds and benchmarking results can be found on Zenodo (zenodo.org/records/14861498). Pangenome reference in a variant calling format (VCF) was downloaded from

https://github.com/human-pangenomics/hpp_pangenome_resources (GRCh38 Graph, Raw VCF). Illumina, PacBio HiFi and Oxford Nanopore data for the HPRC samples can be found at https://s3-us-west-2.amazonaws.com/human-pangenomics/index.html?prefix=working. NYGC variant calls for the 1KGP samples were downloaded from http://ftp.1000genomes.ebi.ac.uk/vol1/ftp/data_collections/1000G_2504_high_coverage/working/20220422_3202_phased_SNV_INDEL_SV. 3202 1KGP Illumina datasets are available on the European Nucleotide Archive under accession codes PRJEB31736 and PRJEB36890.

Research involving human participants, their data, or biological material

Policy information about studies with human participants or human data. See also policy information about sex, gender (identity/presentation), and sexual orientation and race, ethnicity and racism.

Reporting on sex and gender	N/A
Reporting on race, ethnicity, or other socially relevant groupings	N/A
Population characteristics	N/A
Recruitment	N/A
Ethics oversight	N/A

Note that full information on the approval of the study protocol must also be provided in the manuscript.

Field-specific reporting

Please select the one below that is the best fit for your research. If you are not sure, read the appropriate sections before making your selection.

☒ Life sciences ☐ Behavioural & social sciences ☐ Ecological, evolutionary & environmental sciences

For a reference copy of the document with all sections, see nature.com/documents/nr-reporting-summary-flat.pdf

Life sciences study design

All studies must disclose on these points even when the disclosure is negative.

Sample size	Reference panel of 90 haplotypes were used. The size equals the size of the latest (at the moment of submission) HPRC pangenome with 90 phased diploid whole genome assemblies, from where local haplotypes were taken (44 diploid samples + 2 reference assemblies). Direct evaluation was performed for all 40 HPRC samples (80 haplotypes) with available Illumina data; same 40 samples were used for simulated Illumina data. For data storage reasons, long read analysis (PacBio HiFi and ONT) was performed on 20 samples (40 haplotypes). 1KGP call set comparison was performed on all 39 samples with both HPRC assemblies and NYGC diploid calls. Trio concordance was calculated on all 563 trios (1676 samples) from the 1KGP cohort, independent from the HPRC cohort. All available samples were used, except for long read analysis. Sample size of 40 is generally considered sufficient for basic statistical analysis; additionally, any random effects should be almost fully offset by leave-one-out analysis and by large sample-size trio analysis.
Data exclusions	No data exclusion.
Replication	Every samples was analysed twice, with full reference panel and with limited leave-one-out panel to model real life independence between reference panels and analyzed samples. No replications within each analysis was needed since all tools are either deterministic (same analysis produces the same results), or have random elements but produce virtually the same results evrey time. All performed analyses and replications were included in the manuscript or in supplementary information.
Randomization	For long read data, 20 samples were selected randomly. Elsewhere: all available data was used, no allocation needed.
Blinding	Samples were not groupped into case-control, instead the only relevant information could be the similarity between analyzed sample haplotypes and other haplotypes. This information was not used by researchers until the evaluation stage. Furthermore, the analysis was performed automatically using Locityper, which does not support input similarity matrix, and therefore could not be influenced by it.

Reporting for specific materials, systems and methods

We require information from authors about some types of materials, experimental systems and methods used in many studies. Here, indicate whether each material, system or method listed is relevant to your study. If you are not sure if a list item applies to your research, read the appropriate section before selecting a response.

Materials & experimental systems

n/a	Involved in the study
☒	☐ Antibodies
☒	☐ Eukaryotic cell lines
☒	☐ Palaeontology and archaeology
☒	☐ Animals and other organisms
☒	☐ Clinical data
☒	☐ Dual use research of concern
☒	☐ Plants

Methods

n/a	Involved in the study
☒	☐ ChIP-seq
☒	☐ Flow cytometry
☒	☐ MRI-based neuroimaging

Plants

Seed stocks

Report on the source of all seed stocks or other plant material used. If applicable, state the seed stock centre and catalogue number. If plant specimens were collected from the field, describe the collection location, date and sampling procedures.

Novel plant genotypes

Describe the methods by which all novel plant genotypes were produced. This includes those generated by transgenic approaches, gene editing, chemical/radiation-based mutagenesis and hybridization. For transgenic lines, describe the transformation method, the number of independent lines analyzed and the generation upon which experiments were performed. For gene-edited lines, describe the editor used, the endogenous sequence targeted for editing, the targeting guide RNA sequence (if applicable) and how the editor was applied.

Authentication

Describe any authentication procedures for each seed stock used or novel genotype generated. Describe any experiments used to assess the effect of a mutation and, where applicable, how potential secondary effects (e.g. second site T-DNA insertions, mosiacism, off-target gene editing) were examined.
